# Extraction and Characterization of Type I Collagen from Parrotfish (*Scarus sordidus* Forsskål, 1775) Scale solubilized with the Aid of Acetic Acid and Pepsin

**DOI:** 10.1155/2023/7312447

**Published:** 2023-04-26

**Authors:** Abdul Aziz Jaziri, Rossita Shapawi, Ruzaidi Azli Mohd Mokhtar, Wan Norhana Md. Noordin, Nurul Huda

**Affiliations:** ^1^Faculty of Food Science and Nutrition, Universiti Malaysia Sabah, Kota Kinabalu 88400, Sabah, Malaysia; ^2^Faculty of Fisheries and Marine Science, Universitas Brawijaya, Malang 65145, Indonesia; ^3^Borneo Marine Research Institute, Universiti Malaysia Sabah, Kota Kinabalu 88400, Sabah, Malaysia; ^4^Biotechnology Research Institute, Universiti Malaysia Sabah, Kota Kinabalu 88400, Sabah, Malaysia; ^5^Fisheries Research Institute, Batu Maung, Penang 11960, Malaysia; ^6^Faculty of Sustainable Agriculture, Universiti Malaysia Sabah, Sandakan 90509, Sabah, Malaysia

## Abstract

Waste from marine fish processing is an important source of valuable products. Fish collagen is considered a alternative biomaterial due to its excellent properties, and it is widely used for industrial purposes. Thus, this present study aimed to characterize acid and pepsin-soluble collagens from the waste of parrotfish (*Scarus sordidus* Forsskål, 1775) scales. The yields (*p* > 0.05) of acid-soluble collagen (ASC-PFS) and pepsin-soluble collagen (PSC-PFS) were 1.17 g/100 g and 1.00 g/100 g, respectively. Both collagen samples were categorized as type I owing to the presence of two alpha chain subunits (*α*1 and *α*1) after being confirmed by a sodium dodecyl sulfate polyacrylamide gel electrophoresis (SDS-PAGE). Under the fourier transform infrared (FTIR) test, the triple helical structure of type I collagens from the ASC-PFS and PSC-PFS was maintained. Moreover, the study of UV visible spectra and X-ray diffraction (XRD) showed the similarity of collagens derived from different fish species, and the thermostability (*T*_max_) evaluation of all extracted collagens was in the range of 36.22–37.78°C, and their values were comparable to previous research on the fish scale collagens. The effect of various pH and sodium chloride (NaCl) treatments on solubility exhibited that the ASC-PFS and PSC-PFS were highly soluble in an acidic condition (pH < 5.0) and low concentration of sodium chloride (<30 g/L). Taken together, collagens extracted from parrotfish scale waste can be an alternative source for industries.

## 1. Introduction

Collagen is the major structural protein in mammals, representing approximately one-third of the total body protein content. This fiber protein has a specific right-handed triple helical chain containing three parallel polypeptide (left-handed) chains [1]. Typically, it is assembled by a Glycine-Xaa-Yaa triplet, where the Xaa and Yaa positions are usually placed by proline and hydroxyproline [2]. It is ubiquitous in the extracellular matrix (ECM) of tissues, where it not only provides strength and structural stability, but also performs highly specialized regulatory functions, particularly during development and repair [3]. To date, almost thirty types of collagen have been investigated, which differ according to their structure of protein, composition of amino acids and molecular attributes. Amongst them, type I is extensively explored and best studied collagen in the fields of medical, nutraceutical, pharmaceutical, cosmetic, and food application due to its biocompatibility, biodegradability and weak antigenicity [4, 5]. Traditionally, collagen was made from the skin and bones of mammals, such as cows and pigs [1]. However, use of mammalian collagens raises anxiety among consumers, which is related to some infectious diseases, e.g., foot and mouth disease, transmissible spongiform encephalopathy, and bovine spongiform encephalopathy. Also, poultry-based collagens cause fear due to the incidence of the avian flu virus [6, 7]. In another case, some religious groups like Islam and Judaism cannot consume porcine and its derivatives, while bovine is not accepted by Hinduism [8]. In such situations, seafood/fish by-products (skins, frames, and scales) are believed to be potential sources of collagen due to their high availability, low risk of transmittable disease, and free of religious barriers [9].

For a decade, fish collagen has received considerable attention, with the increasing number of studies related to collagen extraction from the fish by-products. In addition, some physicochemical and functional properties of fish collagen have also been elucidated. For instances, purple-spotted bigeye (*Priancanthus tayenus*) skin [10], bigeye tuna (*Thunnus obesus*) skin [11], red stingray (*Dasyatis akajei*) skin [12], sturgeon (*Huso huso*) skin [13], Siberian sturgeon (*Acipenser baerii*) cartilage [14], tilapia (*Oreochromis mossambicus*) bone [15], lizardfish (*Saurida tumbil*) bone [7], Yellowfin tuna (*Thunnus albacares*) swim bladder [16], sardinella (Sar*dinella fimbriata*) scale [17], grey mullet (*Mugil cephalus*) scale [18], sea bass (*Lates calcarifer*) [9], and grass carp (*Ctenopharyngodon idellus*) scales [19]. Although a variety of collagens derive from fish by-products with different species have been documented, other fish sources (especially those generated from processing plants) are required to be investigated in order to give valuable information related to the use of collagen-based products, and it can generate new income and also reduce environmental problems [20, 21].

Parrotfish, also known as “Ikan Bayan” in Malaysia, is a tropical fish species belonging to the family Labridae. Parrotfish (*Scarus sordidus* Forsskål, 1775) is commonly recognized by its parrot-like beak of fused teeth, a bluntly-rounded head, and large scales [22]. It is quite popular with consumers due to its high nutritional content. Parrotfish scale is almost discarded during processing, and to tackle this limitation, transforming it into collagen is a great strategy. Currently, only the skin part of parrotfish has been extracted as collagen, with a high yield obtained [23]. However, other parts (such as the scale) of that fish are much less documented. This study focused on the collagen extraction derived from the scales of parrotfish with the addition of an acid solution and the aid of pepsin enzyme. Their physicochemical properties were also evaluated. Finally, this study could raise the added value of this waste product from fish and be friendly to the environment. Moreover, it may provide some basic information for further studies.

## 2. Materials and Methods

### 2.1. Materials

The scale wastes of parrotfish (*Scarus sordidus* Forsskål, 1775) were provided by a fishmonger in the Kota Kinabalu fish market (Sabah, Malaysia). All fish scales (approximately five kilograms) were packed in a polyethylene bag containing a 2 : 1 (g/g) ratio of ice to scale, and the packed samples were then transported to the laboratory. Upon arrival, the scales of parrotfish were rinsed with running tap water and dried in an electric cabinet dryer (WS340, Tsung Hsing, Kaohsiung city, Taiwan). Acetic acid (100056), Folin–Ciocalteu's phenol reagent (109001), *N, N, N′,N′-*tetramethyl ethylene diamine (TEMED) (110732), Coomassie Blue R-250 (112553), acrylamide (800830), and sodium dodecyl sulfate (SDS) (817034) were supplied from Merck (Darmstadt, Germany). Lowry reagent (L1013), Trizma® hydrochloride (T3253) and bovine serum albumin (BSA) (A3733) were obtained from Sigma Chemical Co., (St. Louis, USA). Precision plus protein dual colour standards (molecular weight markers) (1610374) were supplied by Bio-Rad Laboratories (Hercules, CA, USA). Other reagents and chemicals used in the present study were of analytical grade.

### 2.2. Extraction of Acid- and Pepsin-Soluble Collagen

**Figure 1:**
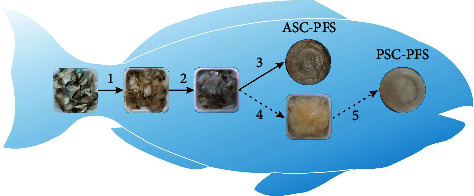
Extraction of parrotfish collagen from scale wastes: (1) NaOH pretreatment, (2) EDTA-2Na pretreatment, (3) acetic acid extraction, (4) residue of acid treatment, and (5) pepsin-assisted extraction.

All extraction processes were exhibited in a cool room (4°C). The method used in this work was slightly modified from the previous research [24–26], as shown in Figure 1. First, dried parrotfish scales (around 50g) were dissolved in 10 volumes (v/w) of sodium hydroxide (0.1 M) and continuously stirred for 6 h, changing the alkaline solution every 2 h to eliminate undesirable pigment and noncollagenous matter. The immersed fish scales were then rinsed with cooled distilled water and neutralized at a neutral condition. Next, 10 volumes of ethylenediaminetetraacetic acid disodium salt (0.5 M) were added to the sample and stirred for 24 h (replacing the solution every 12 h) to demineralize the sample. The demineralized collagens were washed three times with cold distilled water, and then subjected to acid-assisted extraction. A total of fifteen volumes of 0.5 M acetic acid (glacial) were mixed with the pretreated samples and stirred at 500 rpm for 2 days using a homogenizer (IKA® RW 20 Digital Over Head Stirrer, Selangor, Malaysia). After extraction, the suspended collagens were subjected to filtration using a single layer of cheesecloth. The filtrate was collected for a further step, while the residue was kept separately in a freezer for further experimentation (pepsin-aided extraction). Next, the filtrate (soluble fraction) was precipitated by adding 0.05 M Trizma® hydrochloride and 2.5 M NaCl. The precipitated sample was subsequently neutralized (pH 7.0) and then centrifuged for 10 min at 15,000 × *g*. After centrifugation, two volumes of acetic acid (0.5 M) were dropped on the pellet and mixed thoroughly. The liquid extracted collagens were then put into a dialysis tubing. Twenty volumes of acetic acid (0.1 M) and cold deionized water were prepared and dialyzed for 3 days. The dialysate was dried in a Labconco freeze-dryer machine (Kansas City, USA). The dried collagen was represented acid-soluble collagen of parrotfish scale (ASC-PFS). In terms of pepsin-assisted extraction, the residue from previous acid extraction was isolated by adding 15 volumes of acetic acid (0.5 M) and bovine pepsin (Himedia, Maharashtra, India) (1.5%, w/w) for two days. After the isolation process, further procedures were similar to the acid extraction process. The freeze-dried sample was known as a pepsin-soluble collagen of parrotfish scale (PSC-PFS). Both collagen samples were then stored in a freezer until experimentation.

### 2.3. Collagen Analyses

#### 2.3.1. Yield and Colour Analysis

The yield of parrotfish scale collagens was stipulated according to the formula described by Matmaroh et al. [24]

(1) $$\begin{array}{c} Yield\left(\%\right)=\frac{Weight\;of\;died\;collagen\left(ASC\wedge PSC\right)}{Weight\;of\;initial\;dry\;parrotfish\;scale}\times 100\text{.} \end{array}$$

Colour attributes for ASC-PFS and PSC-PFS were assessed through a colorimeter instrument (ColorFlex CX2379, HunterLab, Galveston, TX, USA), as described in a previous study [25]. The differences in the color test were presented in the *CIELAB* or CIE 1976 *L*^*∗*^*a*^*∗*^*b*^*∗*^ colour space, where *L*^*∗*^ is the lightness or brightness, *a*^*∗*^ is the redness (from green to red), and *b*^*∗*^ is the yellowness (from blue to yellow). For whiteness index (WI), both ASC and PSC from the parrotfish scales were determined using a formula stated by Ishamri et al. [27]

(2) $$\begin{array}{c} WI={\left.{\left[\left(100-L^{*}\right.\right]}^{2}+\left({a^{*}}^{2}\right)+\left({b^{*}}^{2}\right)\right]}^{0.5}\text{.} \end{array}$$

#### 2.3.2. UV Absorption Spectrum

Ultraviolet absorption spectra of ASC-PFS and PSC-PFS from the scales of parrotfish (*Scarus sordidus* Forsskål, 1775) were performed under a spectrophotometer of the LAMBDA 25 type (PerkinElmer, Inc., Waltham, MA, USA). A fifty milligram sample was immersed in 10 mL of AcOH solution (0.5 M) and well mixed. Then, the mixture was prepared for centrifugation at 8,500 × *g* for 5 min, and the solubilized collagens were pipetted out into a quartz cell. The spectra of the extracted collagens were designed at wavelengths from 400 nm to 200 nm, and an acetic acid solution with the same concentration was used for a baseline [28].

#### 2.3.3. Attenuated Total Reflectance-Fourier Transform Infrared Spectroscopy (ATR-FTIR)

The ATR-FTIR spectra of the isolated samples (ASC-PFC and PSC-PFC) were analysed using the FTIR spectrometer (Agilent Cary 630, Cary, NC, USA), and all steps were adopted from the study of Matmaroh et al. [24]. The dried parrotfish collagen (20 mg) was placed on the crystal cell of a spectrometer, and the spectra were adjusted between 4,000 nm^−1^ and 800 cm^−1^. All data with significant peaks were observed using a software program developed by Agilent Microlab.

#### 2.3.4. Sodium Dodecyl Sulfate-Polyacrylamide Gel Electrophoresis (SDS-PAGE)

The Mini-PROTEAN Tetra Cell (Bio-Rad Laboratories, Hercules, CA, USA) was used to determine the molecular weight (MW) of the ASC and PSC from the parrotfish scales, with the established methods from Laemmli [29]. A three milligram of each lyophilized collagen was prepared by mixing with a 5% SDS solution and then put in a water bath (at 85°C for 60 min). Afterwards, the treated collagens were centrifuged at 8,500 × *g* for 5 min to eliminate insoluble matter. The supernatant (20 *μ*L) was pipetted into an appropriate centrifuge tube, and then the same volume of sample buffer incorporated, with and without *β*-mercaptoethanol was added. The mixed samples were then heated for 5 min at the same temperature and carefully filled carefully in the prepared acrylamide gel (stacking gel: 4% and separating gel: 7.5%). A certain voltage was set at 120 volts for about 1 h. Next, the fixation process was employed to fix the electrophoresed gel, and it was further stained for about 10 min. After staining, the gel was transferred into a destaining container. The protein marker was used to compare the electrophoretic bands of ASC-PFC and PSC-PFC.

#### 2.3.5. X-Ray Diffraction Analysis (XRD)

Analysis of X-ray diffraction from the parrotfish scale collagen prepared by adding acetic acid and pepsin was conducted according to the previous report [30]. The dried collagen samples were placed into a sample holder and then scanned using an X-ray diffraction machine. The scanning range in both ASC-PFS and PSC-PFS was initiated from 5° to 40° (2*θ*) with a speed of 0.06° per second, and the current and tube voltage of the XRD apparatus were adjusted to 50 mA and 40 kV, respectively.

#### 2.3.6. Differential Scanning Calorimetry (DSC)

The thermal stability test on parrotfish scales was carried out with a Perkin-Elmer differential scanning calorimeter (Model DSC7, Norwalk, CA, USA) under a nitrogen atmosphere. The procedure used in this work was pointed from the study of Kittiphattanabawon et al. [31]. Freeze-dried collagens were prepared for rehydration at a ratio of 1 : 40 (w/v) with distilled water and then incubated for two days in a refrigerator. Afterwards, the prepared samples were accurately weighed (ranging from 5 mg to 10 mg) into an aluminum volatile pan and then tightly sealed with a crimper. Prior to running the samples, a calibration step was performed with an indium. Subsequently, a sealed collagen sample and an empty pan were placed into sample and reference detectors, respectively, and then scanned from 20°C to 45°C at a rate of 1°C per minute. DSC rates were expressed in the maximum transition temperature (*T*_max_) and the total denaturation enthalpy (ΔH).

#### 2.3.7. Solubility Test

Solubility tests at different concentrations of sodium chloride and at various pH treatments were observed in both ASC-PFS and PSC-PFS samples, adopting our previous method [32]. For the sodium chloride assay, the concentrations employed range between 0 g/L and 60 g/L. A total of 5 mL of prepared collagens were pipetted out into five millilitres of sodium chloride (NaCl) solution and then mixed using a stirrer for 60 min in a chiller. Next, the NaCl-treated collagens were subjected to centrifugation at 10,000 × *g* for 15 min to separate insoluble samples. Following centrifugation, the protein content of each parrotfish collagens were analysed based on the established method [33], and a standard protein used in this analysis was bovine serum albumin (BSA). In terms of different pH conditions, both collagens were prepared by dissolving in an 0.5 M acetic acid solution and stirred for 4 h in a chiller. The dissolved collagens were further adjusted at different pH values (pH 1.0–9.0) with adding 1 M NaOH and HCl solutions. Next, the treated collagens were stirred for 60 min and centrifuged further at 10,000 × *g* for 10 min with an Eppendorf centrifuge (Model 5430R, Hamburg, Germany). The protein concentration of each treated sample was also tested with the Lowry method. Both ASC and PSC from the scales of parrotfish were used to determine the percentage of relative solubility using the following formula:

(3) $$\begin{array}{c} Relative\;solubility\left(\%\right)=\frac{Current\;concentration\;of\;protein\;at\;current\;NaCl\;or\;pH}{The\;highest\;concentration\;of\;protein}\times 100\text{.} \end{array}$$

#### 2.3.8. Statistical Analysis

The data in this study were presented as means with SD. The probability value of <0.05 or *p* < 0.05 was defined as a significant difference after being analysed with a one-way ANOVA. To compare the mean values of each treatment, we used Duncan's multiple range tests through SPSS Statistics version 29.0 (IBM Corp., Armonk, N.Y., USA).

## 3. Results and Discussion

### 3.1. Yield and Colour Attributes of Parrotfish Collagens

Table 1 shows the yields of ASC and PSC from the parrotfish scale wastes. Although no significant difference (*p* > 0.05) as observed in both isolated collagen samples, the ASC-PFS showed a higher yield (based on a dry weight basis) than that observed in the PSC-PFS. Both collagens were comparable to the previous study of fish scale collagens. For instances of ASC and PSC from the lizardfish (*Saurida tumbil*) (0.18% and 0.60%) [25], Nile tilapia (*Oreochromis niloticus*) (0.77% and 0.71%) [34], carp (*Cyprinus carpio*) (0.97% and 1.37%) [37], and sea bass (*Lates calcarifer*) (0.38% and 1.06%) [9]. In contrast, the highest yield of fish collagens (either extracted with the acid solution or aided with the pepsin) was obtained from the skin portion, as reported by numerous research studies, such as sturgeon fish (*Huso huso*) (9.98% and 9.08%) [13], sailfish (*Istiophorus platypterus*) (5.76% and 2.11%) [38] and Spanish mackerel (*Scomberomorous niphonius*) (13.68% and 3.49%) [39]. A lower yield in the fish scale collagens could be due to abundant hydroxyapatite components (Ca_5_(PO_4_)_3_OH) and many crosslinked areas of fish scale collagens [40]. Also, the variations in the yields observed in the fish collagens were might be affected by extraction procedures, tissue composition and structure, and fish species with different sizes and ages [41].

**Figure 2:**
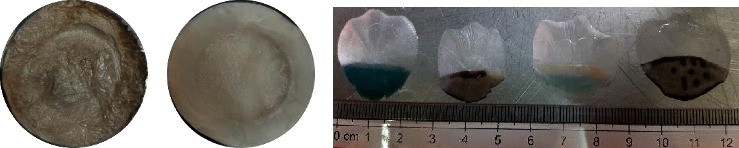
(a) ASC-PFS: acid-soluble collagen from parrotfish scale; (b) PSC-PFS: pepsin-soluble collagen from parrotfish scale; (c) type of parrotfish scales.

In the context of colour attributes, collagen is a potential ingredient supplemented in food, cosmetic, pharmaceutical, and medical products. Hence, collagen with a brighter colour is more acceptable because it does not change the original colour of final products [42]. Table 1 shows the values of *L*^*∗*^, *a*^*∗*^, and *b*^*∗*^, as well as the whiteness index (WI) in both ASC and PSC derived from the parrotfish scales (Figures 2(a) and 2(b)). The results presented indicated that the L^∗^ and WI scores of the PSC-PFS sample were significantly higher (*p* < 0.05) compared to those of the ASC-PFS. Nevertheless, our present study's lightness rate was lower in terms of percentage than that found in the lizardfish (*S. tumbil*) scale collagen [25], snakehead (*Channa argus*) skin collagen treated with hydrogen peroxide (H_2_O_2_) [36], and also type I collagen from calfskin [7]. The reason might be due to the lack of treatment during the decolouration process by adding H_2_O_2_ a solution as prepared in the previous study for snakehead skin collagen, and the type of fish scale used in the experimentation. Typically, parrotfish scales have a colourful pattern, including green, purple, brown, and grey, resulting in the final product obtained, as presented in this study (Figure 2(c)).

### 3.2. Protein Profile

**Figure 3:**
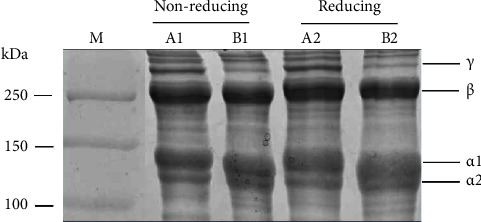
SDS-PAGE profile of ASC-PFS and PSC-PFS. M: protein marker; A1 and A2: ASC-PFS; B1 and B2: PSC-PFS.

Both collagens (ASC-PFS and PSC-PFS) were almost the same in SDS-PAGE patterns (Figure 3) with presenting two alpha chains (*α*1 and *α*2), one *β*- and *γ*-chains. However, there was a slight difference in molecular weight (MW) of *α*1 and *α*2. For the PSC-PFS sample, those chains (*α*1 = 118.1 kDa and *α*2 = 107.4 kDa) were lower compared to those (*α*1 = 123.9 kDa and *α*2 = 112.7 kDa) of the ASC-PFS. Also, the *β*-chain of the ASC-PFS sample showed less band intensity than that of the ASC-PFS sample. These evidences could be due to the fact that some parts of telopeptide regions in terms of crosslinked components were cleaved by the pepsin during the extraction process, as reported by Khittiphattanabawon et al. [34]. In comparison of the band intensity, especially *α*1-chain had a twofold increase over that of *α*2, suggesting that the parrotfish scale collagen was categorized as type I collagen. These obtained data agreed with other fish collagens, including purple-spotted bigeye (*Priacanthus tayenus*) [10], bigeye tuna (*Thunnus obesus*) [11], loach (*Misgurnus anguillicaudatus*) [43], and flying fish (*Cypselurus melanurus*) [18], the golden pompano (*Trachinotus blochii*) [44], and the channel catfish (*Ictalurus punctatus*) [45]. Furthermore, under the treatment of nonreducing (without *β*-ME) and reducing (with *β*-BE), all extracted collagens showed no difference in electrophoretic patterns, indicating no di-sulfide bond formation in both ASC- and PSC-PFC.

### 3.3. UV Absorption Spectrum

**Figure 4:**
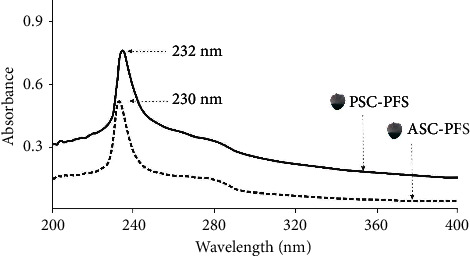
UV absorption spectra of ASC and PSC from the scales of parrotfish.

In general, the significant absorption spectrum of collagen can be observed at a wavelength of 210–240 nm [46]. The obtained results exhibited that the maximum spectra of ASC and PSC derived from the parrotfish scale were detected at wavelengths of 230 nm and 232 nm, respectively (Figure 4). These findings were in accordance with other experiments in fish collagen samples, including lizardfish (*S. tumbil*) [7], Siberian sturgeon (*Acipenser baerii*) [14], and miiuy croaker (*Miichthys miiuy*) [47] and puffer fish (*Lagocephalus inermis*) [48]. Moreover, the absorption peaks depicted in this study (both ASC-PFS and PSC-PFS) are related to the functional groups of collagen molecules, such as carboxyl (-COOH), carbonyl (C=O), and amides (CONH_2_). Meanwhile, for another peak detected in the spectra, there was a low absorption peak at wavelengths of 300−250 nm. These peaks represented tryptophan, phenylalanine and tyrosine (aromatic amino acids), as confirmed in all abovementioned references [25, 26].

### 3.4. Fourier Transform Infrared Spectroscopy (FTIR)

**Figure 5:**
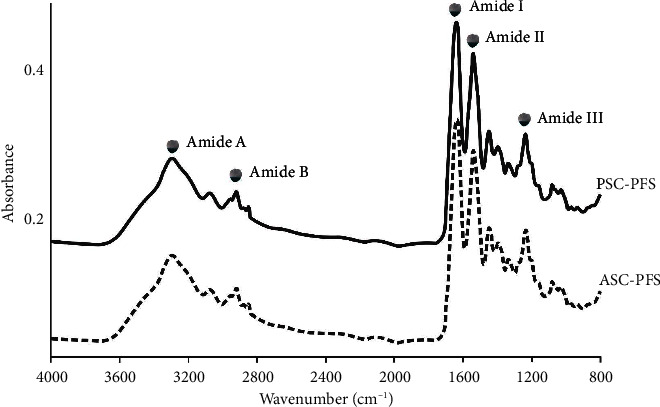
The IR spectra of isolated collagens from the parrotfish scale. ASC-PFS: acid-soluble collagen from parrotfish scale; PSC-PFS: pepsin-soluble collagen from parrotfish scale.

The IR spectra of ASC-PFS and PSC-PFS (Figure 5), and the annotation of each prominent peak in their spectra are also informed (Table 2). In particular, the amides I−III found in both collagens would be applied to assess the triple helical structure of parrotfish scale collagens. According to Nikoo et al. [52], using the formula of Δ*v* (*v*_*I*_ − *v*_*II*_), where the difference in wavenumber (cm^−1^) between amides I and II is less than 100 cm^−1^, suggesting that the triple helical structure of collagen is preserved. After confirmation, both ASC-PFS and PSC-PFS had the same value of delta *v* (∆*v* = 95.05 cm^−1^), and it could be argued that the triple helical structure f the parrotfish scale collagen was maintained. In addition, as proposed by Doyle et al. [49], the triple helical structure of collagen can be verified using an AIII/A1450 ratio, and the result obtained in this study presented that the triple-helix structure obtained from ASC-PFS and PSC-PFS was intact during the extraction process, as also indicated by their absorption ratio values (∼1.0). Taken together, all the prominent peaks of parrotfish collagens are in accordance with the previous reports from lizardfish (*S. tumbil*) scale collagen [25] Nile tilapia (*O. niloticus*) scale collagen [34], carp (*C. carpio*) scale collagen [37], sea bass (*L. calcarifer*) scale collagen [9], and giant grouper (*Epinephelus lanceolatus*) scale collagen [53].

### 3.5. X-Ray Diffraction Analysis

**Figure 6:**
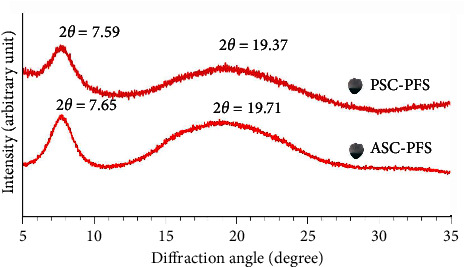
XRD of the parrotfish scale collagens. ASC-PFS: acid-soluble collagen from parrotfish scales; PSC-PFS: pepsin-soluble collagen from parrotfish scale.

The XRD graph of ASC-PFS and PSC-PFS is illustrated in Figure 6. Generally, fish collagen has two significant diffraction peaks, which are sharp and broad peaks, and these peaks were exhibited in our present study. In comparison to both samples, the diffraction peaks were somewhat the same, with the first peak located at 7.65° and 7.59°, and the second peak at 19.71° and 19.37°, respectively. These diffraction peaks were typically found in many previous reports from other fish collagens, such as carp (*C. carpio*) scale [54], tilapia (*O. niloticus*) skin [55], golden pompano (*T. blochii*) skin and bone [44], and lizardfish (*S. tumbil*) skin, bone, and scale [7, 25, 32]. Moreover, to predict the low value of the repetitive spacings,, or *d* (Å), Zhang et al. [54] reported that the Bragg formula could be used in verifying the *d* (Å) through *d* (Å) = alpha/2sin theta in which alpha and theta represent the X-ray wavelength (1.54 Å) and the Bragg diffraction angle, respectively. The *d* value of the first peak was 0.103 Å of ASC-PFS and 0.101 Å of PSC-PFS. This first peak value describes the range within the molecular chains of the triple helical structure in the collagen molecules. For the second peak, the d values of ASC- and PSC-PFS were 0.260 Å and 0.255 Å, respectively, reflecting the spacing of skeletons. These most recent data were consistent with the diameter of a collagen molecule having a triple helical structure and a single left-handed helical chain. Therefore, parrotfish scale-derived ASC and PSC were considered in their native conformations.

### 3.6. Thermal Stability Evaluation

**Figure 7:**
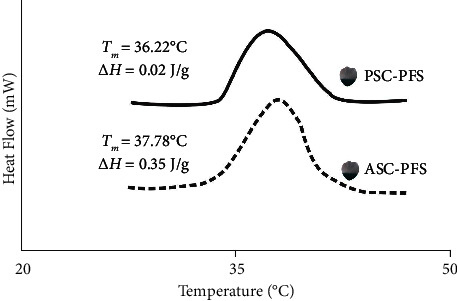
DSC thermogram of the parrotfish scale collagens. ASC-PFS: acid-soluble collagen from parrotfish scales; PSC-PFS: pepsin-soluble collagen from parrotfish scale.

The DSC results for parrotfish scale collagens (*i.e*., ASC-PFS and PSC-PFS) were expressed in the *T*_max_ and Δ*H* values, and the obtained results informed that a higher *T*_max_ (37.78°C) and Δ*H* (0.35 J/g) were recorded in the ASC-PFS sample compared to those of the PSC-PFS (*T*_max_ = 36.22°C and Δ*H* = 0.02 J/g, respectively) (Figure 7). According to Benjakul et al. [56], collagen with a greater *T*_max_ value has greater thermal stability due to the presence of imino acids, especially at pyrrolidine rings located in proline and hydroxyproline that were relatively constructed by H bonding via the –OH group of hydroxyproline, assuming that the imino acids are possibly higher in the ASC-PFS sample than that of the PSC-PFS sample. However, the difference in *T*_max_ values in the fish scale collagens, such as Nile tilapia (*O. niloticus*) (ASC = 36.15°C and PSC = 34.70°C) [34], sea bass (*L. calcarifer*) (ASC = 38.17°C and PSC = 39.32°C) [9], giant grouper (*E. lanceolatus*) (ASC = 35.18–40.86°C) [53] and lizardfish (*S. tumbil*) (ASC = 31.61°C and PSC = 30.54°C) [25, 26] might be depending on amino acids' (particularly imino acids) composition, extraction method, habitat, body temperatures, and collagen conformation [31]. In the context of Δ*H*, PSC-PFS was lower than the ASC-PFS sample, reflecting a lower energy required to uncouple collagen alpha chains and convert them into random turns. It might be due to the cleaved telopeptide area by pepsin in the PSC-PFS sample.

### 3.7. Solubility Study

**Figure 8:**
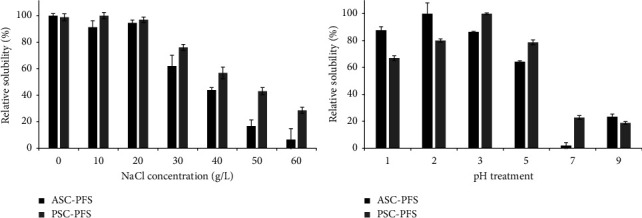
Relative solubility (%) of acid- and pepsin-soluble collagens from parrotfish scales. ASC-PFS: acid-soluble collagen from parrotfish scales; PSC-PFS: pepsin-soluble collagen from parrotfish scale.

Solubility of the ASC-PFS and PSC-PFS samples was tested at different NaCl concentrations and pH treatments, as presented in Figure 8. In both collagens, a highly soluble (>80%) was observed at the NaCl concentration of 0–20 g/L, while the relative solubility of both ASC-PFS and PSC-PFS declined steadily at the concentration of 30 g/L to 60 g/L. It could be due to the high concentration of sodium chloride added during the precipitation process of the solubilized collagen. In addition to this, an increase in NaCl concentration would enhance the interactions of hydrophobic–hydrophobic amino acids within the polypeptide chains of parrotfish scale collagen and at the same time increase competition for water, resulting in the high protein precipitation [30]. Our findings were also supported by other studies on different fish collagens, such as carp (*Hypophthalmichthys nobilis*) scales [57], spotted golden goatfish (*P. heptacanthus*) scales [24], and lizardfish (*S. tumbil*) scales [25]. When comparing those two samples used in this study, the PSC-PFS showed more soluble in almost all NaCl treatments than the ASC-PFS sample. It suggests that the peptide at the telopeptide area cleaved by pepsin might contribute to the solubility of pepsin-soluble collagen from parrotfish scales. In terms of pH treatment, both collagens treated at pH 1.0 to pH 5.0 were soluble, with a relative solubility of more than 60%. The highest solubility was detected at pH 2.0 and pH 3.0 for ASC-PFS and PSC-PFS, respectively. In contrast, under pH 7.0 and pH 9.0 treatments, all collagen samples decreased sharply, representing below 30% of their relative solubility. As reported in numerous works, for instances: lizardfish (*S. tumbil*) scale collagen [25], tilapia (*O. niloticus*) scale collagen [30], horse mackerel (*Trachurus japonicas*), scale collagen and grey mullet (*Mugil cephalus*) scale collagen [18]. It indicates that fish scale collagens, particularly parrotfish scale collagens, are unstable in the neutral and alkaline conditions. The reason could be assumed to be that hydrophobic−hydrophobic interactions occur between the collagen molecules, causing the overall net charge to become zero, especially at the isoelectric point, which usually occurs under neutral conditions [58]. In addition to this, the lowest solubilisation rate was exhibited at pH 7 treatment of ASC-PFS, and it was probably due to the isoelectric point. Overall, the PSC-PFS had higher solubility (particularly under NaCl treatment) because the cleavage of telopeptide areas might affect the protonation of charged amino and carboxyl groups. This could influence the repulsion of molecules associated with the different solubilities [24].

## 4. Conclusion

Type I collagen from the parrotfish scales was successfully extracted by acetic acid (ASC-PFS) and with the aid of pepsin (PSC-PFS). Both samples had a high thermal stability, and the yields were relatively higher compared to some previous studies in the fish scale collagens. Their structure of triple-helix was maintained during the extraction process upon confirmation by the X-ray diffraction and infrared spectroscopy analyses. Thus, collagens from parrotfish scales could be used as an alternative source of collagen for further application.

## Figures and Tables

**Table 1:** Yield and colour parameters of ASC-PFS and PSC-PFS samples.

Sample	Yield (%)	Colour parameters				References
		*L* ^*∗*^	*a* ^*∗*^	*b* ^*∗*^	WI	
ASC-PFS	1.17 ± 0.19^a^	61.74 ± 2.83^b^	2.61 ± 0.05^a^	6.15 ± 0.04^a^	61.16 ± 2.79^b^	This study
PSC-PFS	1.00 ± 0.19^a^	74.81 ± 1.95^a^	1.09 ± 0.24^b^	6.14 ± 0.77^a^	74.44 ± 1.99^a^	This study
ASC-LFS	0.18 ± 0.03	79.94 ± 0.06	1.41 ± 0.15	3.67 ± 0.12	79.56 ± 0.58	[25]
PSC-LFS	0.60 ± 0.06	81.04 ± 0.45	0.17 ± 0.13	11.95 ± 1.34	77.57 ± 0.97	[26]
ASC-NTS	0.77	—	—	—	—	[34]
PSC-NTS	0.71	—	—	—	—	
BMSC	—	65.41 ± 0.08	0.14 ± 0.01	3.16 ± 0.03	65.27	[35]
SHSC	—	89.49 ± 0.28	−0.30 ± 0.01	5.60 ± 0.13	88.09	[36]
CSKC	—	78.93 ± 0.59	−0.07 ± 0.03	1.42 ± 0.27	78.88 ± 0.58	[7]

Values are presented as the mean ± SD from triplicate (*n* = 3). Means provided in the same column with different notation are significantly different (*p* < 0.05). ASC-PFS: acid-soluble collagen from parrotfish scale; PSC-PFS: pepsin-soluble collagen from parrotfish scale; ASC-LFS: acid-soluble collagen from lizardfish scale; PSC-LFS: acid-soluble collagen from lizardfish scale; ASC-NTS: acid-soluble collagen from Nile tilapia scale; PSC-NTS: pepsin-soluble collagen from nile tilapia scale; BMSC: barramundi skin collagen; SHSC: snakehead fish skin collagen; CSKC: type I collagen from calfskin.

**Table 2:** The prominent peak region and its description for ASC-PFS and PSC-PFS.

Peak area		Peak description	References
ASC-PFS (cm^−1^)	PSC-PFS (cm^−1^)		
3286.67	3297.85	Amide A: N-H stretching coupled with hydrogen bond	[49]
2427.90	2926.97	Amide B: CH_2_ asymmetric stretching	[50]
1636.34	1636.34	Amide I: C=O stretching/H bond coupled with COO-	[51]
1541.29	1541.29	Amide II: N–H bend coupled with C-N stretching	[51]
1234.71	1235.64	Amide III: N–H bend coupled with C-H stretching	[51]

ASC-PFS: acid-soluble collagen from parrotfish scale; PSC-PFS: pepsin-soluble collagen from parrotfish scale.

## Data Availability

Data used to support the findings of this study are available from the corresponding author upon reasonable request.
